# Revisiting therapeutic hypothermia for severe traumatic brain injury… again

**DOI:** 10.1186/cc13955

**Published:** 2014-06-30

**Authors:** Donald W Marion, Lemma E Regasa

**Affiliations:** 1 The Defense and Veterans Brain Injury Center, 1335 East–west Highway, Suite 605, Silver Spring, MD, 20910, USA

## Abstract

Improved understanding of the molecular mechanisms of secondary brain injury has informed the optimum depth and duration of cooling and led to increased clinical interest in the therapeutic moderate hypothermia for severe traumatic brain injury over the past two decades. Although several large multi-center clinical trials have not found a treatment effect, multiple single-center trials have, and a recent meta-analysis by Crossley and colleagues now finds that the cumulative findings of those single-center trials dilute the multi-center trial results and show an overall reduction in mortality and poor outcomes associated with cooling. The need for consistent support of key physiologic parameters during cooling is emphasized by this finding.

## Introduction

A recent meta-analysis of clinical trials of the use of hypothermia to treat severe traumatic brain injury (TBI) is the first report in nearly 15 years to suggest improved outcomes associated with cooling [1]. Therapeutic moderate hypothermia (32 to 35°C) for the acute care of patients with severe TBI has been evaluated in multiple clinical trials during the past 20 years, especially in the US, Japan, and China. A review of 11 prospective randomized clinical trials that compared intracranial pressure (ICP) levels with hypothermia versus normothermia treatment and six prospective cohort studies that provided ICP data before and during hypothermia treatment found that therapeutic moderate hypothermia was surpassed only by hypertonic saline, lumbar cerebrospinal fluid drainage, and decompressive craniectomy in the level of ICP reduction [2]. There are several mechanisms through which cooling may reduce swelling following an injury, including a reduction of pro-inflammatory cytokine levels, decreasing the cellular inflammatory response, and stabilization of the blood–brain barrier. Pre-clinical brain injury studies have consistently found reduced lesion volume, reduced inflammation, and improved functional outcomes with post-injury cooling. Beneficial effects on these mechanisms are consistent with the apparent efficacy of hypothermia for neonatal hypoxic-ischemic encephalopathy and pre-hospital cardiac arrest. Indeed, therapeutic moderate hypothermia is now recognized as the clinical standard of care for these two diseases.

## Prospective multicenter trials have not found benefit

Enthusiasm for the treatment of patients with severe TBI with moderate hypothermia was significantly dampened by a large prospective randomized clinical trial (published in 2001) that did not find any benefit with the treatment compared with a normothermia control group. Subsequent Cochrane meta-analyses of all clinical trials of hypothermia for TBI also could not discern an overall benefit. In fact, a pediatric multi-center trial actually suggested an increased mortality rate in the hypothermia group compared with the control group.

The findings of the meta-analysis by Crossley and colleagues [1] are therefore surprising and unexpected. The study appears to be methodologically sound: they appropriately tested the level of bias and heterogeneity among studies they included in the analysis, and they provided a separate analysis of data from studies with a low risk of bias. Both the cumulative and ‘low risk of bias’ data sets showed significant positive effects of hypothermia. The authors used relative risk (RR) as their measure of the analysis. Outcome events (death and poor outcomes such as long-term disability) in the TBI population are not rare events and, under such a scenario, RR tends to be biased and odds ratios are preferred by some. However, since the authors are not actually comparing the odds of developing an outcome (death) among groups (gender, age group, and so on) with respect to their exposure (hypothermia), the simplest and easiest measure (RR) is acceptable.

There is an apparent distinction between single-center versus multi-center trials, however, and this has been observed by others [3]. In the report by Crossley and colleagues, 17 studies described mortality rates in the hypothermia and normothermia groups that included at least 10 patients in each group. The three multi-center studies - Clifton and colleagues (2001 [4] and 2011 [5]) and Maekawa (2009) [6] - found either no difference or worse mortality rates in the hypothermia groups, but all of the 14 single-center studies found that therapeutic hypothermia was associated a lower mortality rate. Likewise, for clinical studies with 10 or more subjects in the treatment and control groups, there were significantly fewer poor outcomes in the hypothermia groups, but only in the single-center studies and not in the multi-center studies. A significant variation in critical care across sites was reported for the NABIS: H (National Brain Injury Study: Hypothermia) multi-center study and could confound hypothermic treatment effects [4]. For example, frequent occurrences of hypotension or hypovolemia in some of the centers of the multi-center trial could result in an increased incidence of poor treatment-related outcomes that could dilute the good treatment-related outcomes seen in the other centers. One could argue that the experimental treatment cannot be very robust if it cannot be shown to be beneficial in a multi-center trial which more closely resembles the real world.

Two large pediatric multi-center clinical trials were excluded from this analysis, but there is no clear evidence that the hypothermia effect should be substantially different for children than it is for adults. If the pediatric studies by Adelson and colleagues [7] and Hutchison and colleagues [8] had been included, the meta-analysis may not have found a benefit, particularly with the latter study finding a significant increase in mortality in the hypothermia group.

Clinical trials of therapeutic moderate hypothermia for severe TBI are still ongoing. The Eurotherm 3235 Trial is an international, multi-center, randomized controlled trial which is examining the effects of titrated therapeutic hypothermia (32 to 35°C) and is coordinated by Peter Andrews at Western General Hospital, Edinburgh, and has enrolled more than 300 of the targeted 600 patients. Clifton (personal communication) has organized a new randomized clinical trial to look just at those patients with surgical intracranial mass lesions. Maekawa [9] completed a multi-center study of 150 subjects in 2008 and has presented preliminary data at several meetings.

## Conclusions

In addition to endorsing focused treatment based on the type of cranial injury, most believe it is important to cool the patient to target temperature as quickly as possible, preferably within 4 to 6 hours after the injury. Intravascular cooling technology to allow such rapid cooling and to better maintain the patient at target temperature currently exists. With more rapid cooling and better selection of the candidates for this therapy, it is possible if not likely that future multi-center trials will find the therapeutic efficacy that single-center studies already have found. However, it is clear that there will need to be close monitoring of the acute care of these patients to ensure consistency of support of blood pressure, intravascular volume, and other aspects of medical management across the centers.

## Abbreviations

ICP: Intracranial pressure; RR: Relative risk; TBI: Traumatic brain injury.

## Competing interests

The authors declare that they have no competing interests.

## Authors’ information

The views expressed in this commentary are those of the authors and do not necessarily reflect official views or policy of the US Army, the US Department of Defense or Veterans Affairs, or the US Government.
